# N-Alkanes in Permafrost Peatlands

**DOI:** 10.3390/plants14030449

**Published:** 2025-02-03

**Authors:** Alexander Pastukhov, Dmitry Kaverin, Sergey Loiko

**Affiliations:** 1 Institute of Biology Komi Science Centre Ural Branch Russian Academy of Sciences, Kommunisticheskaya 28, 167982 Syktyvkar, Russia; dkav@mail.ru; 2 BIO-GEO-CLIM Laboratory, National Research Tomsk State University, Lenina 36, 634050 Tomsk, Russia

**Keywords:** permafrost, peatlands, hydrocarbon, degradation, warming, biomarker, organic carbon

## Abstract

In this study, high-performance liquid chromatography (HPLC) methods were utilised to identify and quantify C21–C33 n-alkanes in permafrost peatlands located within the Eastern European and Western Siberian cryolithozone. The total content of n-alkanes in Europe is 7.4 times higher compared to Siberian permafrost peatlands, and was estimated at 282 ± 145 (range from 74 to 709) μg/kg and 38 ± 12 (10–66) μg/kg, respectively. In the European cryolithozone, CPI alkane 9.5 ± 2.4 (3.7–18.6) and HPA 0.10 ± 0.03 (0.05–0.23) indicate a relatively higher share of higher plants and a higher stage of peat decomposition decree, with 6.9 ± 2.1 (3.1–12.9) and 0.15 ± 0.05 (0.06–0.29) in the Siberian region. In contrast, the Western Siberian peat plateaus were formed under conditions of constant excess moisture, a distinction from the Eastern European peatlands, where moisture and permafrost conditions were subject to constant change. This is further corroborated by the values of Paq, C23/C29 and C23(C27 + C31), which are 0.90 ± 0.05 (0.69–0.99); 11.1 ± 8.9 (0.84–61.6); 1.53 ± 0.80 (0.21–4.72) and 0.47 ± 0.12 (0.08–0.71); 0.64 ± 0.32 (0.08–1.48); and 0.43 ± 0.21 (0.04–1.26), respectively. The n-alkanes and peat physicochemical properties show no significant correlation. In the European part, permafrost degradation occurred repeatedly during the warming periods. Nevertheless, only slight subsidence of the permafrost table was observed, and peat continued to accumulate (up to 0.1 mm/year) in the West Siberian peat plateaus. Consequently, the variation in the quantitative and qualitative composition of n-alkanes in permafrost peatlands is determined not only by the different botanical composition of the plant remains forming the peat strata, but also by the consequence of lower mean annual temperatures in Western Siberia compared to the European nNortheast, and such a climatic difference persisted throughout the Holocene.

## 1. Introduction

Peatlands play an important role in the cycling of carbon and nitrogen [1]. Northern peatlands alone occur 3.7 ± 0.5 million km^2^, and store 415 ± 150 Pg and 10 ± 7 Pg of nitrogen [2]. It is estimated that approximately 50% of the area of peatlands is in a permafrost. It is therefore vital to acknowledge the vulnerability of these peatlands to permafrost degradation. Permafrost peatlands store fragile carbon stocks vulnerable to warming because thaw-induced thermoerosion can drive peatland inundation, which strengthens methane emissions [3,4]. The conservation of northern peatlands as a carbon sink and stock is crucial to achieving net-zero global CO_2_ emissions and mitigating climate warming by 2050 [5]. However, it should be noted that peatlands also emit CH_4_ into the atmosphere [6]. Thus, different conditions for peatland genesis and peatland vegetation, even within one bog complex, lead to changes in the carbon balance [7]. The dynamics of CO_2_ and CH_4_ exhibit significant variation in bryophyte plant communities due to the influence of vascular plants [8]. Consequently, when conducting paleoreconstruction, it is essential to consider the varying conditions of peatland genesis [9].

It is evident that the climatic period sequence has been documented in ice cores, marine sediments and peat samples [10]. Conventional methods (the study of the macrofossil remains and palynological composition of peat) have been utilised to analyse subarctic and arctic ecosystems. However, there is an increasing demand for new qualitative approaches in order to gain a more profound understanding of how these ecosystems are going to respond to climate change. Recently, novel geochemical methods have been developed that reveal functional characteristics and bioindicators of peat properties [11,12,13,14]. The geochemical analysis of the peat composition has revealed that the lipid fractions of plants contain biomarkers, i.e., specific chemical compounds that identify both individual plants and groups of plants that make up the peat strata [15,16]. One of these biomarkers is the ratio of n-alkanes, which themselves are resistant to degradation and quite reliably indicate the degree of decomposition of organic carbon and the species composition of plant communities of peat strata [17,18,19,20].

Concurrently, the majority of studies analyse biomarkers for high-moor peatlands, while there is a paucity of information regarding the functionality of these biomarkers in transitional and low-moor peatlands, where peat undergoes a higher degree of decomposition and humification. Some studies utilise the same biomarkers even when the peatland consists of high-moor and transitional/low-moor peat [15]. However, recent research [21,22] has demonstrated that the concentrations and ratios of n-alkanes in mosses and select vascular plant species characteristic of low-moor peatlands differ, even when the same mosses and plants are present in high-moor peatlands. As in high-moor peat [17], in transition/low-moor peat, the predominance of sphagnum is labelled by medium-chain-length alkanes and the terrestrial parts of vascular plants by long-chain alkanes. However, the results showed similar concentrations of n-alkanes in sphagnum and underground parts of sedges in low-moor peat. Consequently, using the n-alkane ratios known for upland peat for transition/low-moor peat would lead to misinterpretations about the actual proportions of these plant groups in peat [21]. Analogous disparities in the concentrations of medium-chain-length n-alkanes are present in the roots of vascular plants [23].

There has been only sporadic use of n-alkane analysis to monitor environmental change in permafrost peatlands [13,19,20,24,25]. This study evaluated the qualitative and quantitative composition of n-alkanes in relation to the morphological and physicochemical characteristics of permafrost peatlands in the East European and West Siberian Plains.

## 2. Materials and Methods

### 2.1. Site Description

**Figure 1:**
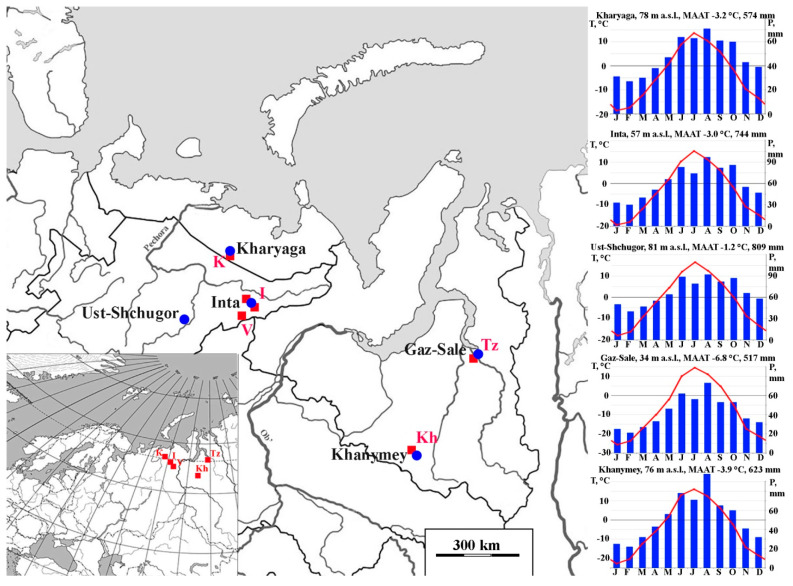
The map illustrates the location of the closest climate stations, indicated by blue circles, and the sampling sites used for the study, represented by red squares. The following diagrams illustrate the mean monthly precipitation and air temperatures, based on the climate database (https://ru.climate-data.org/ (accessed on 24 September 2024)).

The selected study area encompasses the permafrost peat plateau/thermokarst complexes (polygonal and uplifted mounds and fens) of the European Northeast and Western Siberia, corresponding to the extent of the tundra and the northern taiga ecoclimatic zones within underlined continuous, discontinuous and isolated permafrost (Figure 1). The mean annual air temperature in the lowlands varies from −1.2 °C in the southwest to −6.8 °C in the northeast, with mean annual precipitation ranging from 517 to 809 mm (of which 2/3 falls in the summer months, May to October [26]. The orographic effect of the Ural Mountains increases the precipitation in the European and decreases it in the Siberian part of the study area. The topography of the lowlands is predominantly flat, with elevations ranging from 40 to 160 m a.s.l. These plains are underlain by substantial quaternary glacial and lacustrine deposits, which collectively create the primary topographic features [26]. The flat mesorelief and shallow permafrost table facilitate waterlogging. The landscape is characterised by the prevalence of deep river valleys, ravines, shallow thermokarst streams and lakes, which are indicative of the significant role played by water in the region’s geomorphology. The eolian and abrasion processes are predominantly observed along steep slopes, riverbanks and lake shores, underscoring the dynamic nature of the landscapes.

Two peat sections (TZ and K) were collected from two permafrost peat plateau/thermokarst complexes in the southern tundra, while the remaining three sections (Kh, V and I) were located in the northern and extreme northern taiga, respectively. Polygonal peatland with polygonal-roller soil complexes TZ—“Tazovsky”: TZ 1, TZ 2 and TZ 3—67°20′ N, 78°56′ E) was studied in the southern tundra with continuous permafrost in Western Siberia. Permafrost peatlands of peat plateaus with shallow permafrost tables interspersed with permafrost-free fens and thermokarst lakes open or in-filled with vegetation were investigated in the northern taiga of Western Siberia, in the area known as Kh—“Khanymei”: Kh 2 and Kh 3—63°42′ N, 75°54′ E, and in the European Northeast, in the southern tundra (K—“Kolva”: K 1 and K 2—63°42′ N, 75°54′ E) and in the extreme northern taiga (I—“Inta”: I 1—65°54′ N, 60°26′ E; I 2—65°25′ N, 60°49′ E; I 3—66°04′ N, 60°05′ E; I 4—66°00′ N, 60°05′ E; I 11—66°05′ N, 59°58′ E). The soils of polygons and peat plateaus are classified as Ombric Sapric Cryic Histosols (Hyperorganic), which are characterised by the presence of well-decomposed organogenic material (peat) extending to a depth of more than 2 m deep, predominantly atmospheric nutrition and the underlying permafrost within 1 m (following the World Reference Base for Soil Resources terminology) [27]. The soils of the interpolygonal cracks belong to Hemic Muusic Histosols, where the moderately decomposed peat accumulates on the polygonal-veined ice filling the cracks. The soils of seasonally freezing fens are identified as Hemic Histosols. The presenst of permafrost is either absent or located deeper than 10 m in fens (the maximum depth of our borehole drilling). The soils of the peat circles (bare surfaces on permafrost peatlands) are classified as Ombric Sapric Cryic Histosols (Hyperorganic Turbic), as the upper horizons exhibit indications of peat cryoturbation. These bare peat circles are characterised by circular patches with a diameter ranging from 4 to 25 m and an area spanning from 10 to 500 m^2^, covering up to 1–10% of the total peatland.

The raised peat mounds (which can reach heights of 2–3 m) are drier than the peat circles and have a sparse vegetation cover. In contrast to palsas, the peat mounds do not have isolated frozen cores, as they are extensions of the permafrost peat plateau. The mounds are often oval in shape, elongated in the northwest–southeast direction by dozens of metres. The polygonal peatland is characterised by a relatively flat topography, with an excess of polygons over depressions (cracks) reaching up to 0.5 m.

Consequently, the majority of the peat platform is underlain by permafrost: from the Tazovsky site with an extensive continuous permafrost 250–450 m thick with mean annual ground temperature (MAGT), varying from −3 to −7 °C and a significant distribution of syngenetic sediments with a volumetric ice content up to 40–60%, and a shallow active layer (<0.5 m), where open taliks are only found under the channels of large rivers and deep lakes, to the Inta site with sparse island permafrost that is 0–25 m thick and MAGT between 0 and −0.5 °C [28,29].

### 2.2. Soil Sampling and Laboratory Analyses

The peat soils were described and sampled in the centre of peat mounds and polygons, as well as in adjacent fens and interpolygonal cracks. Soil pedons were sampled at intervals of 5 to 10 cm. In the majority of the sections, the reference sampling depth corresponded to full-peat strata. The peat samples were collected in August 2015 and 2017. At Seida, Inta and Kolva sites, deep boreholes (10 m) were drilled and soil samples were collected using a machine tool UKB 12/25–02 “Pombur” in November 2015.

The peat samples were then subjected to analysis at the Chromatography Joint Access Center of the Institute of Biology (Komi Science Center, Ural Branch of the Russian Academy of Sciences). An accelerated solvent extraction system, the ASE-350 (Dionex Corporation, Sunnyvale, CA, USA), was used to extract n-alkanes from the peat samples. The triturated peat sample (1 g) was placed into the extraction cell and subjected to three extractions with a 1:1 methylene chloride–acetone mixture at 100 °C and 1650 psi. The extracts were then concentrated in a Kuderna–Danish apparatus (t = 70 °C); the solvent was replaced with hexane. The resulting concentrate (V = 2 cm^3^) was purified from the polar organic impurities using column chromatography with silica gel 60 (Fluka 60741; particle size, 0.063–0.2 mm) as a sorbent. The n-alkanes were measured by gas chromatography in a TRACE DSQ (Thermo Scientific, Waltham, MA, USA) mass spectrometer in a total ion current mode. The identification of the hydrocarbons was facilitated by the Xcalibur Data System (version 1.4 SR1) and the NIST05 library of mass spectra (version 2.0, containing 220,000 compounds). The quantitative analysis was conducted by measuring the three ions with masses of 57, 71, and 85, which are characteristic of n-alkanes, using dodecane C_12_H_26_ as the internal standard.

A standard mixture of n-alkanes, a blank sample, and a replicate sample were added to each batch of the samples (maximum, 10 samples) in order to test for the impurities, quantitative detection, identification of peaks, and the precision and accuracy of the method. It was established that any target compounds were undetectable in the blank sample. The metrological characteristics of the method (n = 5, *p* = 0.95) were assessed when analysing the C21–C33 n-alkanes (with an odd number of carbon atoms). The quantification of individual n-alkanes was conducted by the internal standard method. The measurement error of n-alkanes was found to be 10–20% for *p* = 0.95.

The analytical data were processed using the Microsoft^®^ Excel 2010 software package and a statistical package R for data processing in ecology [30]. To study the relationship between PAHs and indicators of the peat physicochemical properties, a matrix of similarities was performed based on the Pearson correlation coefficients.

### 2.3. N-Alkanes’ Indicess

Some n-alkane indices reflect their accumulation and distribution in the peat strata and can be used to paleoreconstruct the conditions of peat genesis for which they are calculated.

The average chain length index (ACL) is a measure of the predominance of high- vs. low-molecular-weight n-alkanes.

(1) $$\mathrm{ACL}=\frac{\sum {\left({n*C}_{n}\right)}_{\mathrm{odd}}}{\sum {\left(C_{n}\right)}_{\mathrm{odd}}},$$

In order to assess the transformation of bioproducers of organic matter, the carbon preference index (CPI_alkane_) was calculated, which is the ratio of odd and even alkanes [31]. The CPI index was found to exhibit elevated values in higher plants; however, in organic matter buried in sediment, due to diagenesis and microbial input, it decreases over time, approaching one in mature deposits and oil, as well as in bacteria and algae [32].

(2) $${\mathrm{CPI}}_{\mathrm{alkane}}=\frac{\sum {\left(C_{21}-C_{31}\right)}_{\mathrm{odd}}+\sum {\left(C_{23}-C_{33}\right)}_{\mathrm{odd}}}{2\sum {\left(C_{22}-C_{32}\right)}_{\mathrm{even}}},$$

Terrestrial plants contain the predominance of n-C22–C30 alkanols and are dominated by even chain moieties [33]. CPI_alkanol_ has been shown to exhibit a strong even abundance, with implications for climate change research [34].

(3) $${\mathrm{CPI}}_{\mathrm{alkanol}}=\frac{\sum {\left(C_{20}-C_{26}\right)}_{\mathrm{even}}+\sum {\left(C_{22}-C_{28}\right)}_{\mathrm{even}}}{2\sum {\left(C_{21}-C_{29}\right)}_{\mathrm{odd}}},$$

The higher plant alkane (HPA) index has been shown to serve as a quantitative metric for the degradation of saturated vs. functional compounds [35]. As n-alkanes are known to be preserved preferentially in comparison to n-alkanols, a decline in the HPA index is indicative of an escalated rate of decomposition [36].

(4) $$\mathrm{HPA}=\frac{{\left(C_{24}+C_{26}+C_{28}\right)}_{\mathrm{alkanol}}}{{\left(C_{24}+C_{26}+C_{28}\right)}_{\mathrm{alkanol}}+{\left(C_{25}+C_{27}+C_{29}\right)}_{\mathrm{alkane}}},$$

The humidity index, P_aq_, is utilised to facilitate the reconstruction of the hydrological characteristics of peatlands. This index is based on the predominance of C23 and C25 n-alkanes in submerged and floating macrophytes, and C29 and C31 in land plants and algae [37].

(5) $$P_{\mathrm{aq}}=\frac{C_{23}+C_{25}}{C_{23}+C_{25}+C_{29}+C_{31}},$$

The C23/C29 ratio has been utilised as an indicator of paleohydrological conditions. The divergent n-alkane chain length distributions observed between Sphagnum and other peat-forming vascular plants offer a means to estimate the relative contributions of these plants to peat. In their study, Nichols et al. adopted the C23/C29 ratio for computing the relative abundance of Sphagnum and other plants in the study [17].

The ratio C23/(C27 + C31) has also been employed to reconstruct paleohydrological conditions, particularly in the Eastern European forest-tundra [38].

Shifts in the n-C23/n-C25 ratio have been shown to track changes in the abundance of *Sphagnum fuscum* (specifically the C25 n-alkane) in the macrofossil record [11].

## 3. Results

### 3.1. The Temperature Regime of Permafrost Peatlands

Permafrost peatlands comprise an alternation of peat plateaus (frozen peat mounds/polygons) of various heights and shapes with fens (watered depressions) and lakes. A significant disparity exists between the quantity and quality of water supply in peat plateaus and fens. Thin fens and interpolygonal cracks are mineratrophic; that is, they receive not only atmospheric, but also ground nutrition from the underlying mineral deposits. Typically, fens and the cracks and depressions that intersect them are dominated by a Shrub–Eriophorum–Sphagnum phytocenosis, comprising species such as *Salix* spp., *Eriophorum × medium* Andersson and *Rubus chamaemorus* L., as well as *Vaccinium microcarpum* (Turcz. ex Rupr.) Schmalh. The ground layer is characterised by the presence of *Sphagnum subsecundum* Nees, *S. riparium* Angstr., *S. compactum* Lam. and DC., and brown mosses (*Warnstorfia* spp.). Despite the relatively thick peat deposit of the peat plateaus, they are poor in mineral nutrition, due to their position above the surface of the bog massif, which results in their receipt of only ombrotrophic (atmospheric) nutrition. Mound and polygon vegetation varies throughout the region under study, but polydominant shrub–lichen communities are common, e.g., *Betula nana* L., *Ledum palustre* L., *Vaccinium vitis-idaea* L., *V. uliginosum* L., *Empetrum hermaphroditum* L., *Rubus schamaemorus* L., Bryidae mosses (e.g., *Dicranum* spp., *Polytricum* spp., *Pleurozium schreberi* Mitt) and lichens (e.g., *Cladonia* spp.).

**Figure 2:**
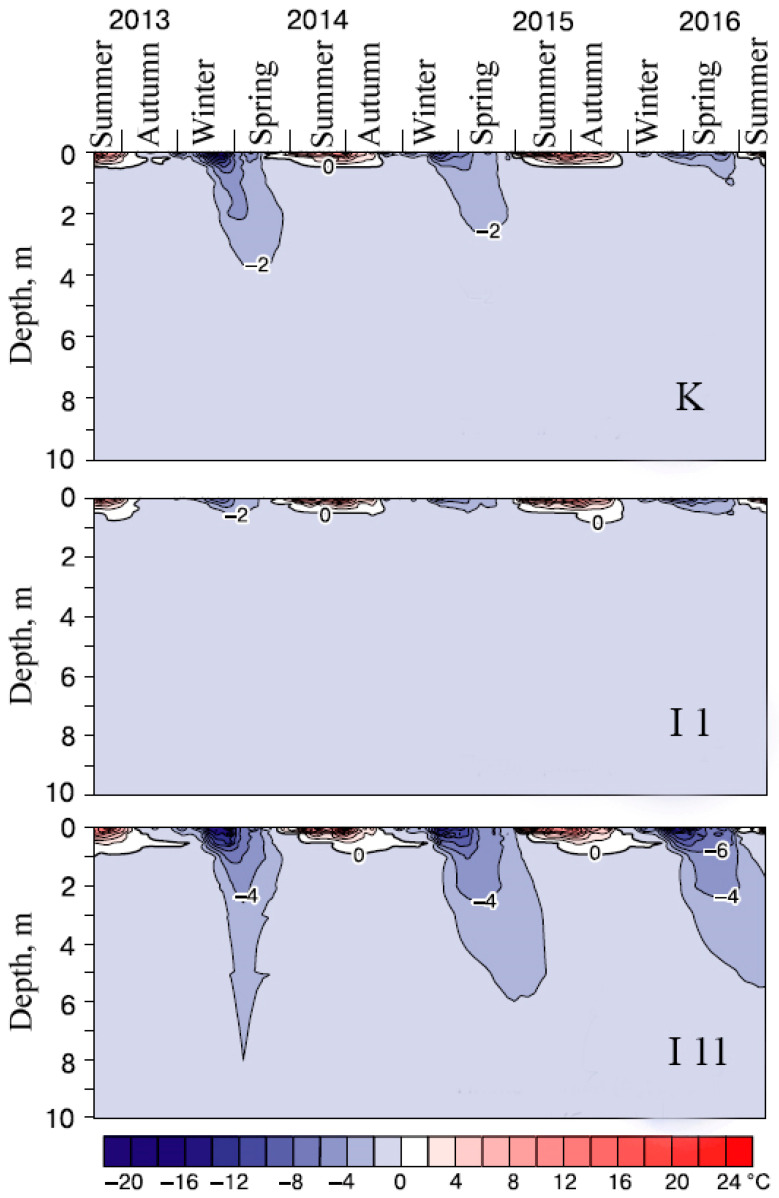
Temperature chronoisopleths of soils and the underlying deposits in peat plateaus K, I 1, and I 11 during 2013–2016.

The present study of the modern temperature regime of peat plateaus showed that if seasonal freezing of the surface horizons of peat mounds commence in October–November, periods of near-zero temperatures are to be expected at depths of 20 cm for a duration of up to 2 months (Figure 2) [39]. However, the presence of a thin snow cover (10–44 cm) during winter, coupled with the increased thermal diffusivity of the high-ice horizons characteristics of peat plateaus, enables the accumulation of a sufficient supply of cold, thereby contributing to the additional cooling of permafrost horizons. The shrub–moss cushion serves as an effective heat insulator during summer, and even on peat circles (patches devoid of vegetation with low albedo, up to 10%), the upper layer of peat undergoes desiccation, thereby facilitating slow summer thawing. Consequently, these factors contribute to the preservation of permafrost, thus enhancing the stability of peat plateaus as ecological systems [40].

**Figure 3:**
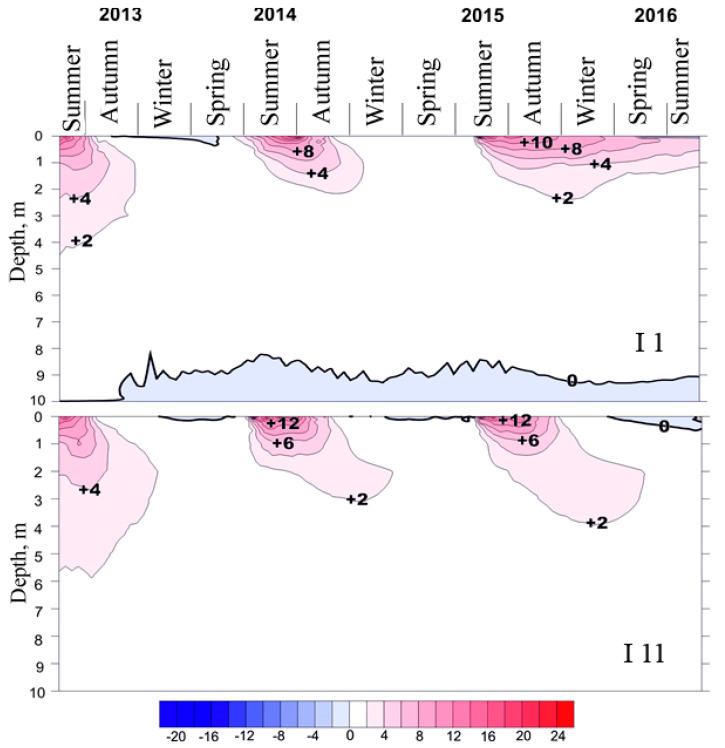
Temperature chronoisopleths of soils and the underlying deposits in fens I 1 and I 11 during 2013–2016.

The temperature regime of fens differs considerably from that of peat plateaus. Due to the strong redistribution of snow and the presence of a thick snow cover (90–120 cm), winter freezing occurs much later and to a lesser extent. In the southern cryolithozone, even at a depth of 20 cm, only positive mean monthly temperatures can remain, at +0.1 °C (Figure 3) [39]. The temperatures of the Fibric Histosols (fen soils) in summer are significantly higher than those of the Cryic Histosols (peat plateau soils), which is explained by the significant water content of the fens, and the absence of or a much weaker cooling effect of permafrost.

### 3.2. General Physicochemical Characteristics of the Studied Peatlands

Modern studies have revealed significant differences in the organic matter of peat strata in the active layer and permafrost in composition and age, which indicate different degrees of resistance to changes in temperature and peat mineralization [19].

**Figure 4:**
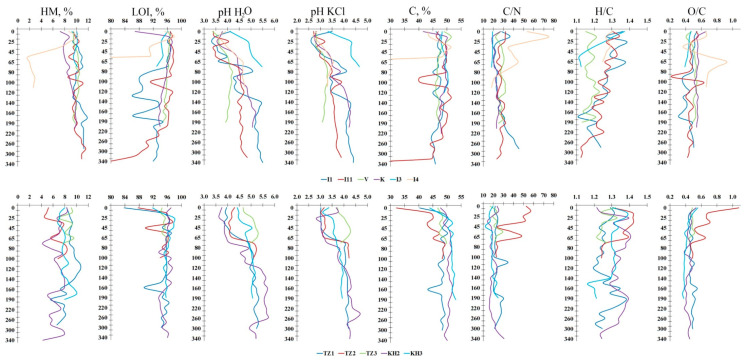
Physicochemical properties of the studied peat plateaus. HM, %—humidity moisture; LOI, %—loss on ignition.

A comparison of the physicochemical properties of peat deposits from East European bogs and West Siberian permafrost peatlands was undertaken, resulting in the identification of significant differences (Figure 4). Specifically, the hygroscopic humidity exhibited significant variation, ranging from 1.7 to 11.8%, with an average value of 9.6 ± 0.8%, in the European Northeast (EN), and noticeably in a narrower range, from 4.2 to 10.7%, with a mean of 7.7 ± 1.1%, in West Siberia (WS). The estimates of loss of ignition are 94.4 ± 2.2% and 95.2 ± 1.2%, respectively. Despite the lower pH H_2_O and pH KCl values of 4.5 ± 0.5 and 3.6 ± 0.5 of the European peatlands, compared to 4.8 ± 0.5 and 3.8 ± 0.4 for the Siberian peatlands, an increase in pH was observed as the profile was traversed. Soil organic carbon ranged from 40.5 to 51.5%, with a mean of 47.8 ± 1.7%, and from 32.1 to 53.1%, with a mean of 49.1 ± 2.0%, at EN and WS, respectively. The presence of weakly decomposed wood residues in the peat strata were identified as the primary factor contributing to the minimum carbon values observed.

Significant variations in median nitrogen concentrations and C/N ratios were observed across different peatland strata. Nitrogen values ranged from 0.79 to 4.9%, with a mean of 2.49 ± 0.50%, while the median C/N ratios in peat across European and Siberian peatlands ranged from 18 to 75 and 12 to 57 (depending on depth); the median peatland categorical values ranged from 27 ± 5 to 23 ± 5, respectively. The broad range in peat C and N values (and C/N ratios) among these bogs is attributable to numerous factors, including variations in vegetation composition, litter decomposition rates [41], acidity [42] and peat mineral content. The high C/N ratio of the peat can be attributed to three primary factors. Sphagnum, the dominant plant in bogs, exerts a significant influence on the low nitrogen (N) values and high C/N ratios characteristic of these ecosystems [43]. Northern permafrost peatlands are distinguished by their notably decomposition rates [44] and microbial activity [45]. The low C/N ratio is attributed to the advanced stage of decomposition when nitrogen is enriched to carbon, minerotrophic peat, and the prevalence of peat circles (unvegetated bare peat surface) with very high nitrous oxide (N_2_O) emissions [46] lead to the low C/N ratio. The accumulation rates of carbon and nitrogen are affected by environmental changes associated with permafrost aggradation. During the Holocene epoch, in tundra peatlands, syngenetic permafrost aggradation has been observed to decrease decomposition through the freezing of relatively undecomposed organic matter, thereby increasing C/N ratios and carbon accumulation rates [47]. Furthermore, frost heave has been observed to affect the peat strata, rendering them vulnerable to winter abrasion caused by wind-drifted ice crystals, which have the potential to remove the ombrotrophic peat, exposing the fen-origin peat to the surface [48]. The C/N ratio in the near-surface bog peat ranges from 24 to 75, indicating high initial vegetation ratios.

The highest C/N ratios were detected in the unfrozen near-surface horizons (0–20 cm) with a fresh undecomposed moss–shrub litter (sites I 4 and TZ 2), with the exception of peat circles (sites I 3 and TZ 3) and/or eroded upper peat layers (site V), which display a low C/N ratio. In the lowermost part of the active layer and the transition layer (45–75 cm), the C/N ratio undergoes a precipitous decline to 12 to 29 before exhibiting a further increase to 16 to 45 throughout the lowermost profile (Figure 4). The transition layer is defined as the layer between the active layer and permafrost, which can presumably thaw in some warm years. In permafrost, the C/N ratio gradually increases down the profile, except in horizons with multiple weakly decomposed remains of shrub–tree vegetation.

The H/C atomic ratios vary from 1.1 to 1.4, with a mean of 1.2 ± 0.1 at the European peatlands, and a range of less 1.2–1.4, with a mean of 1.3 ± 0.1 at the West Siberian bogs. These indicate the predominance of aliphatic chains in the molecules of humic acids, in the presence of 40–50% aromatic structures. The oxidation state (O/C) is estimated at 0.5 ± 0.1 (0.2–0.9) and 0.5 ± 0.1 (0.4–1.1), respectively, indicating the predominance of oxidation processes within the active layer.

The observed differences in physicochemical properties are indicative of changes in paleogeographic environments during the genesis of the studied peat plateaus. Specifically, a flat peat plateau (site I 1) was initially dominated by eutrophic herbaceous-hypnotic communities, characterised by a prevalence of hygrophilic species. Subsequently, it was covered by mesotrophic shrubs, and finally, by hygrophilic eutrophic species. This sequence is indicative of the dynamic shifts in environmental conditions, which can lead to significant variability in the physicochemical properties of peat across strata. In the foothill peat plateau (site I 11), the preponderance of mesoeutrophic herbaceous communities was observed at all stages of its development [49], as evidenced by the lower carbon content, pH, and atomic elemental ratios H/C and O/C, along with a significantly higher degree of humus enrichment with nitrogen, ash content of peat, and C/N ratio.

### 3.3. Concentrations and Distribution of N-Alkanes in Peatlands

The formation of peat plateaus is predominantly influenced by periglacial dynamics, which in turn impact the fundamental and molecular geochemical composition of peat. N-Alkanes, defined as specific chemical compounds demonstrating resistance to degradation, can be utilised to decipher the species composition of peat-forming vegetation. In the studied peatlands, the identification of C14–C31 n-alkanes revealed that long-chain homologues (>C21) with an odd number of carbon atoms in the molecule predominate. The most prevalent in the waxes of higher plants are C27, C29 and C31, while in mosses and sphagnum, C23 or C25 predominate [50,51]. The presence of a limited number of hydrocarbons with an average chain length of C14–C20 molecules indicates that n-alkanes from algae and bacteria contribute insignificantly [52]. The total content of n-alkanes in EN permafrost peatlands is almost an order of magnitude higher compared to WS peat plateaus, averaging at 282 ± 145 in the range from 74 to 709 μg/kg and 38 ± 12 (10–66) μg/kg, respectively.

N-alkanes, higher alcohols and carboxylic acids are synthesised by living organisms and contain an even number of carbon atoms. The decarboxylation of higher alcohols and carboxylic acids, results in the shortening of the carbon chain by one atom, leading to the formation of odd n-alkanes within peat. However, under reducing conditions, the reduction reaction predominates without altering the number of carbon atoms in the chain, and in this case even n-alkanes will be synthesised [53]. The total amount of “even” alkanes in all peatland horizons is several times higher than the number of odd ones, with a value of 28.5 ± 14.8 (5.5–76.7) μg/g in EN and 5.4 ± 2.1 (1.1–15.7) μg/g in WS permafrost peatlands.

**Figure 5:**
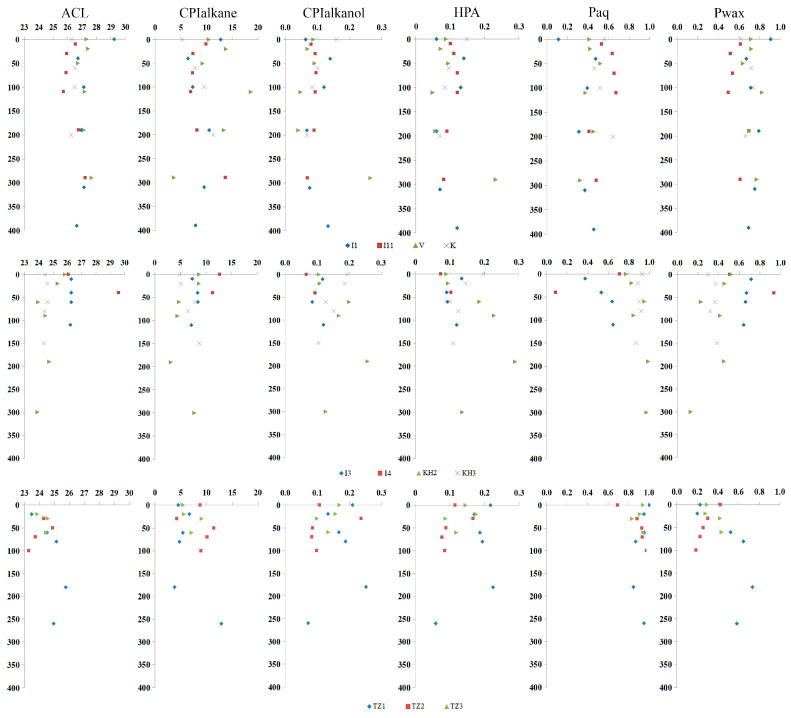
The index values of the studied peat plateaus.

Consequently, the accumulation and distribution of n-alkanes in the peat strata are utilised for paleoreconstruction of peat genesis conditions, for which a number of indices are calculated (Figure 5).

The average carbon chain length (ACL) index is significantly higher in the European Northeast (EN), compared to Western Siberia (WS), and averaged 26.8 ± 0.6 in the range of 25.7–29.6 and 24.5 ± 0.5 (23.3–25.8), respectively.

The carbon preference index CPI_alkane_ is also exhibits significant variation between EN and WS permafrost peatlands, with an average of 9.5 ± 2.4 (3.7–18.6) and 6.9 ± 2.1 (3.1–12.9), respectively.

The CPI_alkanol_ value is 0.10 ± 0.03 (0.04–0.26) in EN and 0.15 ± 0.04 (0.07–0.26) in WS permafrost peatlands. Concurrently, the highest values are predominantly observed in the lowermost part of the peat strata, suggesting the long-term prevalence of reducing conditions.

The HPA value of 0.10 ± 0.03 (0.05–0.23) in EN permafrost peatlands indicates a comparatively higher degree of peat decomposition compared to 0.15 ± 0.05 (0.06–0.29) in WS bogs.

It is evident that the WS permafrost peatlands were formed under conditions of constant excess moisture, in contrast to the EN bogs, where moisture conditions were constantly changing. This is evidenced by the values of P_aq_, C23/C29 and C23(C27 + C31), which are 0.90 ± 0.05 (0.69–0.99), 11.1 ± 8.9 (0.84–61.6), 1.53 ± 0.80 (0.21–4.72) and 0.47 ± 0.12 (0.08–0.71), 0.64 ± 0.32 (0.08–1.48), 0.43 ± 0.21 (0.04–1.26), respectively.

Shifts in the n-C23/n-C25 ratio have been shown to track changes in the abundance of *Sphagnum fuscum* (specifically the C25 n-alkane) in the macrofossil record [11]. The ratio has been found to support the application of n-alkane biomarkers in peat archives for tracking past shifts in the abundance of individual *Sphagnum* species. The values of the C23/C25 ratio are 1.03 ± 0.45 (0.33–2.35) in the EN and 1.31 ± 0.32 (0.33–1.96) in the WS permafrost peatlands.

### 3.4. The Relationship Between the N-Alkane Concentrations in the Studied Peatlands

**Figure 6:**
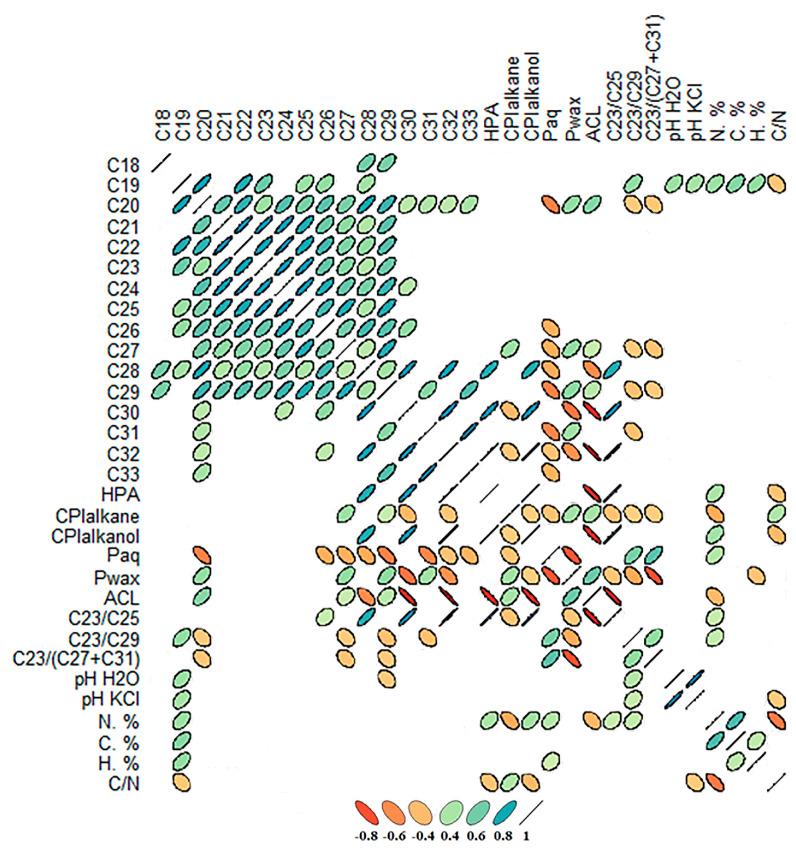
The coefficients of Pearson correlation between the n-alkanes and the main indicators of peat physicochemical properties.

A correlation analysis was performed, and a matrix of Pearson correlation coefficients was constructed to study the interdependence between individual n-alkanes, their indices (HPA (higher plant alkane), P_aq_ (humidity index), P_wax_ (wax index), CPI_alkane_ and CPI_alkanol_ (carbon preference index), ACL (average chain length)) and ratios (C23/C29, C23/C29 and C23/(C27 + C31)), with some physicochemical peat properties (Figure 6).

The ACL demonstrates a strong correlation with the content of C31, C33 and C29 (R = 0.80, *p* ˂ 0.01; 0.64 and 0.63, *p* ˂ 0.05, respectively). The ACL also depends on the concentration of Paq and Pwax indices (R = 0.95 and 91%, *p* < 0.01, respectively) (Figure 6). A strong positive average correlation was identified between the amount of C30 and C23 (R = 0.90, *p* ˂ 0.01), as well as between C21 (R = 0.82, *p* ˂ 0.01). Furthermore, a significant positive correlation was found between C29 and C20 (R = 0.75, *p* ˂ 0.05).

No statistically significant correlations were identified between the n-alkanes and the physicochemical properties of peat. Weak positive relationships were recorded between C19 and the content of C, H, N, and pH H_2_O and pH KCl values, which are 0.54, 0.50, 0.46, 0.46 and 0.42, respectively. Conversely, a negative yet very weak correlation was identified between C29 and pH H_2_O (R = –0.31, *p* < 0.1). Weak correlations of the some indices (HPA, CPI_alkane_, CPI_alkanol_ and ACL) are total nitrogen (R = 0.40, –0.49, 0.45 and –0.41, respectively, *p* < 0.05).

## 4. Discussion

### 4.1. Temperature Regime of Permafrost Peatlands During the Holocene

The composition of peat, including its lipid part, as well as the species of peat-forming plants, is contingent on the hydrothermal and climatic conditions that prevailed during the formation of the peat strata. As previously referenced, peat plateaus and fens exhibit marked differences in water supply quantity and quality, and temperature regime. Consequently, the qualitative composition of peat is influenced by disparate environmental conditions, with fen peat exhibiting reduced acidity and advanced decomposition (humification) in comparison to peat plateaus [54]. Furthermore, due to the natural succession of peat plateaus to ombrotrophic conditions, both as a result of an increase in peat thickness and as a result of cryogenic heaving, layers of minerotrophic lowland peat are deposited in a significant part of the peat strata of mounds and polygons. Consequently, during the Holocene epoch, in periods of warming, contemporary peat plateaus were susceptible to complete degradation, transforming into contemporary fens. Their hydrothermal regime exhibited similarities to that of modern fens or aapa-bogs.

Paleoreconstructions demonstrate that substantial peat accumulation in the studied peatlands commenced during the Preboreal stage, a period characterised by lower temperatures and humidity levels compared to contemporary conditions. A notable decline in peat accumulation, reaching a state of near-complete cessation, occurred in numerous regions by the mid-Atlantic, concurrent with the ongoing climatic optimum [49,55]. In contrast to the tundra and forest-tundra of the European Northeast, where peat accumulated in unfrozen bogs covered with woody plants, sedges and mosses, which began to freeze at the beginning of the Subboreal stage (4600–4300 BP), after which the degradation of permafrost occurred repeatedly during warming periods. In stark contrast to the aforementioned conditions, the Western Siberian tundra experienced a distinct climatic phenomenon. During the period of maximum warming attributed to the Atlantic optimum climate, the region underwent a decline in temperature, yet this did not result in a substantial lowering of the permafrost table. The upper horizons within the tundra maintained an ongoing accumulation of peat, reaching a rate of up to 0.1 mm per year. According to S. Fotiev [56], even in the southern Yamal and Gydan regions, the “heads” of ice wedges are positioned directly below the modern base of the active layer. This indicates that the climate remained stable severe even during warming periods in the Holocene epoch.

### 4.2. The Factors of the Non-Uniform Distribution and Very Wide Range of the N-Alkane Concentrations in the Studied Peatlands

There are many potential variations in the distribution of n-alkanes in the peat profiles studied. In EN peat plateaus the maximum proportions of total alkanes and concentrations are C27, C29 and partly C31, reflecting the contribution of cuticular waxes from higher woody vascular plants [57]. Conversely, in WS permafrost peatlands, only C27 alkanes exhibit a significant proportion, while C29 and C31 alkanes predominate in high-moor peat, suggesting the prevalence of shrub species on the peat plateaus and sedge-shrub vegetation on the adjacent fens [10]. In contrast, the proportion of shrub vegetation in the peat strata of WS peatlands is considerably lower.

The presence of only minute quantities of short-chain C20 n-alkanes suggests a minimal contribution from bacteria and algae [58]. Despite the considerable variation in the values observed, the proportion of C23 and C25 n-alkanes is nevertheless significant in the permafrost peatlands under study. While published data indicate that C23 and C25 n-alkanes are associated with hygrophilic vegetation, particularly sphagnum [50], it should be noted that Sphagnum as a peat former is not exclusive to lowland peat, but is also present in high-moor peat [49].

The concentrations of long-chain hydrocarbons, particularly C25, C27 and C29 n-alkanes, exhibit a marked increase in certain horizons at several key sites. Furthermore, these concentrations do not appear to be influenced by the peat localization in the active layer or in permafrost, or in the so-called transition layer, which in some years is in a permafrost state, and in extremely warm years can thaw [59]. A noticeable decrease and change in concentration of long-chain alkanes appears only in the lower part of the peat strata located directly above the mineral deposits. The absence of discernible trends in hydrocarbon concentrations within both the surface active and deeper permafrost layers of the peat indicates a remarkably constrained organic carbon decomposition, both during the Holocene Optimum and in the contemporary era. The predominance of anaerobic conditions, a phenomenon observed in both the moist environments of lowland peatlands during the Holocene Optimum (a characteristic of EN peatlands) and at sub-zero temperatures within permafrost (where permafrost exhibited no degradation during the Holocene Optimum in WS peatlands), has been identified as the primary factor hindering decomposition. The observed shifts in n-alkane concentrations can be attributed to alterations in vegetation. This finding is consistent with our earlier observations of an absence of trends in changes in C/N and *δ*^13^C [10]. However, a notable trend is the decrease in the content of n-alkanes and the increase in the degree of peat decomposition, despite the identical botanical composition. When interpreting the reasons for changes in the n-alkane concentration, it is necessary to take into account the different rates of plant decomposition, i.e., mosses decompose more slowly than vascular plants [60].

Across a range of vegetation types and key study sites in EN permafrost peatlands, a general trend of decreasing overall n-alkane concentration with soil depth has been observed. This decrease is attributed to the degradation of n-alkanes by soil microbiota. The transformation of n-alkanes into n-methyl ketones occurs through subterminal oxidation [61], while n-alcohols are produced through terminal oxidation, which can subsequently be converted into aldehydes and ultimately into n-fatty acids [62]. Conversely, n-alkanes can also be produced in situ through the decarboxylation of n-fatty acids [61]. When subsequently focusing on the genesis of the n-alkanes with depth in soil profiles is subsequently focused upon, the trends are generally in line with the expected microbial degradation over time. Tomas et al. found a nearly ubiquitous trend of a decreased total concentration of n-alkanes either with time in litterbag experiments or with depth in open plant–soil systems [63].

Nevertheless, an intriguing case is formed by our study of Siberian peat profiles, which reported increasing concentrations of n-alkanes with a depth. M. Keiluweit et al. [64] proposed that under (partially) anaerobic conditions, reduced organic compounds such as lipids and waxes could be selectively protected from degradation. This would result in a relative increase in the n-alkane concentrations with time and, consequently, also with depth until a certain threshold in anaerobic environments such as peatlands. However, even the minimum total content of n-alkanes in the EN permafrost peatlands is higher than the maximum amounts of n-alkanes found in the West Siberian ones (74 and 66 μg/kg, respectively). Earlier studies, e.g., [57], demonstrated in such peat sequences that the historical progression of peatland development, influenced by changing biogenic inputs, can exert a more pronounced influence on n-alkane concentrations than the selective enrichment under anaerobic conditions as proposed by M. Keiluweit et al. [64]. The latter may be more pertinent to nearly steady-state conditions of Histosols, characterised by the continuous accumulation of similar peat biomass over extended periods. Previous studies have shown that the accumulation and retention of n-alkanes in temperate and boreal raised bogs is highly dependent on climate change [65]. Furthermore, it has been demonstrated that the saturated hydrocarbon content is determined not only by the different botanical composition of plant macrofossils forming the peat strata, but also by the temperature and humidity conditions during the Holocene in permafrost peatlands from the tundra to the northern taiga in both the European Northeast [19,20] and Western Siberia [54].

**Figure 7:**
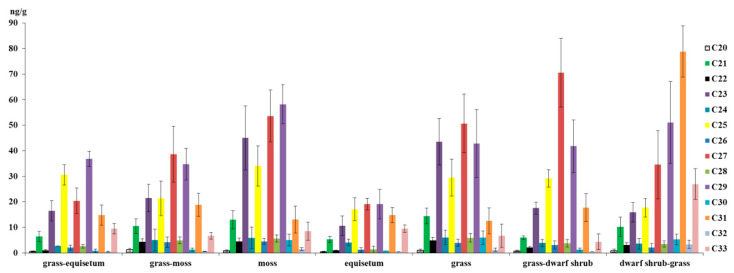
The distribution of n-alkanes in peat samples of diverse plant macrofossils. The error bars represent the standard deviation from the mean (generally n = 3).

The present study posits that the quantitative and qualitative composition of n-alkanes is contingent on the plant macrofossils and trophicity of the peatlands (Figure 7).

It is evident that dwarf shrub–grass peat exhibits the highest concentration of n-alkanes, characterised by a pronounced predominance of long-chain hydrocarbons, specifically C27–C33, with C31 being of particular significance. Conversely, in all other peat samples from different plant macrofossils, the proportion of C31 is minimal, suggesting a negligible contribution to the composition of peat-forming plants associated with drier conditions, such as *Rhododendron tomentosum* (*Ledum palustre*) and various grasses: Poaceae grasses, Juncaceae rushes and Ericaceae heathers [66]. However, homologues of C31 and C33 n-alkanes were not identified, despite the plant macrofossils of the studied WS peat state comprising dwarf shrubs, including *Rhododendron tomentosum* (*Ledum palustre*), as well as Poaceae and Ericaceae.

In grass-dwarf shrub peat, the amount of n-alkanes is 21% less and their distribution is radically different. In comparison with dwarf shrub-grass peat, the dominant homologue in grass-dwarf shrub peat is C27, which is associated with woody plants [16]. Indeed, the analysis of plant macrofossils indicates the presence of the macroremains of *Betula*, *Salix* and *Pinus sylvestris* [49].

Significant quantities of C23 and C25 n-alkanes have been recorded in moss peat, indicating the predominance of Sphagnum mosses. This assertion is corroborated by the analyses of plant macrofossils [49,55]. Concurrently, the presence of Bryidae mosses is indicated, albeit to a minor extent [66]. However, these are present in minimal quantities within the botanical composition of the studied peat strata. The botanical composition of peat is generally heterogeneous, and moss peat often contains a fairly large proportion of higher herbal plants. It has been established that C27–C29 homologs predominate in *Eriophorum vaginatum* L., *Scheuchzeria palustris* F.Muell. and certain species of sedge [10,16]. This observation potentially provides a rationale for the observed similarity in the n-alkane distribution across moss, grass, and grass–moss peats, despite the significantly lower total n-alkane levels observed in the latter.

The minimum amount of n-alkanes is noted in equisetum peat; this is due to much lower concentrations of C23–C25 and C27–C29 homologues compared to peats of other plant macrofossils. In grass–equisetum peat, the proportion of C25 and C29 notably increases due to the significant presence of *Scheuchzeria palustris* and sedges (Cyperáceae), resulting in the sum of all homologues of saturated hydrocarbons being almost 1.5 times higher compared to equisetum peat.

**Figure 8:**
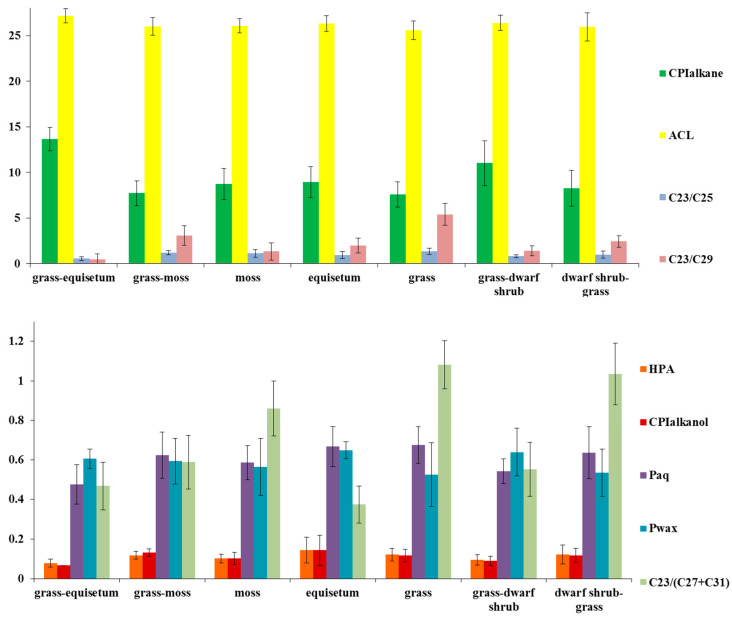
The n-alkane ratio distribution in peat samples of diverse plant macrofossils. The error bars represent the standard deviation from the mean (generally n = 3).

The average carbon chain length index ACL for all peat types ranges from 25.6–27.2 (Figure 8), while the carbon preference index CPI varies from 7.6 ± 1.4 to 13.4 ± 1.3. The minimum CPI values are typical of grass and grass-moss peat, while the maximum is observed in grass-dwarf shrub and grass-equisetum peat. It was previously determined that sphagnum mosses have minimum ACL and CPI values, 25–26 and 4.3–6.9 in most samples, respectively. The CPI in herbaceous plants ranges from 4.8 to 9.0, while in heather shrubs the CPI is 10.7 in chamedaphne and 36.0 in crowberry [66]. However, as previously mentioned, the average ACL and CPI values in WS are lower than in EN permafrost peal and even lower than the minimum values typical for grass peat. The lowest values of the ACL index and the highest values of the humidity index Paq were recorded for samples of high-moor peat in the northern taiga, forest-tundra and tundra in Western Siberia, i.e., areas with the lowest mean annual air temperature (MAAT) [66]. Consequently, within the scope of our study, the lower values of ACL and CPI are contingent not on the species of peat-forming plants, but rather on the consequences of lower MAATs in Western Siberia in comparison to the European Northeast. This climatic disparity persisted throughout the Holocene epoch.

High CPI_alkane_ values are indicative of well-preserved organic carbon [67,68]. In all the permafrost peatlands that have been the focus of this study, CPI_alkane_ values are very high, with the sum of odd alkanes being many times higher than the sum of even alkanes. This indicates the abundance of vascular plants in the peat strata [58]. Concurrently, the concentration of odd alkanes demonstrates a clear independence from peat type, with comparable maximum and minimum values recorded in high-moor, transitional, and lowland peat. The maximum values of odd alkanes are found in peat with the lowest decomposition degrees, indicating extremely low degradation of organic carbon, a finding consistent with the studies of other authors [67]. The minimum CPI_alkane_ values are observed at the contact of peat with mineral deposits, which can be explained by the fact that anaerobic conditions were not immediately established at the initial stages of peat accumulation, and the peat organic carbon became more mineralized. The upper 70–80 cm of most peatlands demonstrate greater variability in the CPI_alkane_ indices and the sum of n-alkanes. The lowest CPI_alkane_ values, which are indicative of favourable conditions for microbial degradation [33], are characteristic of peat plateaus with eroded upper peat layers (sites I 3, V, TZ), the majority of which are in the form of bare peat circles (devoid of vegetation cover). Conversely, other peatlands, predominantly vegetated by dwarf shrubs and dicranous mosses, exhibit high CPI_alkane_ values, indicative of relatively dry conditions. In the lower permafrost part of the peat strata, CPI_alkane_ values mark changes in hydrogeological conditions that occurred during the Holocene, which is in good agreement with our studies of the plant macrofossil and palynological composition [40,49].

The minimum values of C23/(C27 + C31) 0.37 are indicative of equisetum peat, while the maximum is attributed to the high proportion of sedge Cyperáceae—1.04 and 1.08 for grass and dwarf shrub–grass peat, respectively. Furthermore, the ratios C23/C25 and C23/C29 reach their maximum values of 1.35 and 5.39 in grass peat, while in grass–equisetum peat, these ratios are minimal, with values of 0.54 and 0.45, respectively. Equisetum is capable of survival in dry conditions, though it is generally found in waterlogged areas. Consequently, it can be deduced that the prevalence of equisetum peat signifies alterations in environmental conditions, characterised by frequent periods of low temperature and humidity, during the formation of peat strata.

The distribution of other n-alkane indices is approximately equal, and their dependence on the plant macrofossil composition of peat is weak. In accordance with the findings of previous studies [57,69], high values of the P_aq_ index (>0.5) indicate a significant contribution to the botanical peat composition by submerged and floating macrophytes under conditions of excess moisture. This interpretation has been utilised in paleo-reconstructions of non-permafrost raised bogs [65]. However, it is important to note that sphagnum mosses have the capacity to thrive in drier conditions and can also contribute significantly to C25 alkanes [51]. This suggests that the P_aq_ ratio may be subject to bias. The peat layers of the key sites under study contain a mixture of mosses and vascular plants in varying proportions, which makes the interpretation of paleohydrological conditions in our case, using only P_aq_ indices, very inaccurate.

High values of the wax index, P_wax_, are indicative of a greater contribution of vascular plants, which in non-permafrost raised bogs is associated with colder and drier conditions [33]. The predominance of higher vascular plants is indicated by relatively high values (≥0.7) of P_wax_, as observed in sites I 1 and V. In the upper peat layers, individual P_wax_ values reach 0.8 or more, and P_aq_ decrease less than 0.4, suggesting the predominant contribution of woody dwarf shrubs and shrubs [38]. These plants grow/grew in relatively dry conditions on peat plateaus.

## 5. Conclusions

It is important to note that the sums and indices of n-alkanes, which themselves are resistant to degradation, are quite high-quality geochemical markers of the natural environment changes during the Holocene. This is because they reliably indicate the decomposition degree of organic carbon and the botanical composition of plant communities from which peatlands were formed. However, it is important to note that n-alkane ratios cannot be relied upon as the sole reliable marker tool for permafrost peatlands. The permafrost aggradation is accompanied by ice segregation, leading to the uplift of previously swampy lowland peat layers. These processes contribute to increased decomposition of organic carbon in the active layer, since peat, previously located in the anoxic catotelm, begins to be affected by aerobic degradation in the acrotelm. All these changes complicate the correct interpretation of markers, so it must be taken into account that previously obtained and published conclusions on identified markers for non-permafrost boreal peatlands cannot always be used as such for peat plateaus located in permafrost conditions.

In Eastern European permafrost peatlands, the maximum shares in the n-alkane total amount are C27, C29 and, in some cases, C31, reflecting the contribution of shrub vegetation on peat plateaus and shrub-sedge vegetation in fens. In contrast, in Western Siberia, only C27 alkane has a significant proportion. The presence of a limited number of short-chain C20 n-alkanes indicates the prevalence of bacteria and algae. Conversely, a substantial proportion of C23 and C25 alkanes is indicative of the prevalence of hygrophilic vegetation. In European peatlands, a discernible trend is evident: there is a decrease in n-alkane content concomitant with an increase in the degree of peat decomposition, and this is observed for the same plant species. This phenomenon can be attributed to the degradation of n-alkanes by soil microbiota. Conversely, an increase in n-alkanes has been observed in Western Siberia, attributable to the persistent maintenance of anaerobic conditions. Notably, even the minimum total content of n-alkanes in Eastern European peat plateaus exceeds the maximum of n-alkane sum recorded in Western Siberia (74 and 66 μg/kg, respectively).

The CPI_alkane_ 9.5 ± 2.4 (3.7–18.6) and HPA indices 0.10 ± 0.03 (0.05–0.23) in East European permafrost peatlands indicate a relatively higher proportion of higher plants and a high degree of peat decomposition in comparison with West Siberian with 6.9 ± 2.1 (3.1–12.9) and 0.15 ± 0.05 (0.06–0.29), respectively. West Siberian peat plateaus were formed under conditions of constant excess moisture, in contrast to East European permafrost peatlands, where moisture conditions were subject to constant change. This is corroborated by the values of P_aq_, C23/C29 and C23(C27 + C31), which are 0.90 ± 0.05 (0.69–0.99); 11.1 ± 8.9 (0.84–61.6); 1.53 ± 0.80 (0.21–4.72) and 0.47 ± 0.12 (0.08–0.71); 0.64 ± 0.32 (0.08–1.48); 0.43 ± 0.21 (0.04–1.26), respectively.

There are no significant correlations between the n-alkanes and peat physicochemical properties.

In contrast to the East European plain, where permafrost degradation repeatedly occurred during warming periods, in the West Siberian tundra even the maximum warming of the Atlantic optimum climate occurred within negative temperatures and did not lead to a significant lowering of the permafrost table. Furthermore, the upper peat horizons continued to accumulate at a rate of up to 0.1 mm per a year. The content of saturated hydrocarbons in permafrost peatlands from the tundra to the northern taiga in both the European Northeast and Western Siberia is determined not so much by the species of peat-forming plants, but due to lower mean annual temperatures in Western Siberia compared to the European Northeast, and the like climatic difference persisted throughout the Holocene.

## Data Availability

The datasets used during the current study are available from the corresponding authors on reasonable request.
